# Supplementary material for: Intervening Effects and Molecular Mechanism of Quercitrin on PCV2-Induced Histone Acetylation, Oxidative Stress and Inflammatory Response in 3D4/2 Cells
Source: Antioxidants (Basel). 2022 May 11;11(5):941. doi: 10.3390/antiox11050941 (PMC9137775; doi:10.3390/antiox11050941)
Supplement: Supplementary file 1 [file antioxidants-11-00941-s001.zip › antioxidants-1671903-supplementary.pdf]

Table S1 Gene sequences

Gene name	Sequence
β-actin	F:5'-GATGAGATTGGCATGGCTTT-3'
	R:5'-CACCTTCACCGTTCCAGTTT-3'
Nrf2	F:5'-GAAAGCCCAGTCTTCATTGC-3'
	R:5'-TTGGAACCGTGCTAGTCTCA-3'
Gpx1	F:5'-GGCACAACGGTGCGGGACTA-3'
	R:5'-AGGCGAAGAGCGGGTGAGCA-3'
Keap1	F:5'-GCCTCATCGAGTTCGCTTAC-3'
	R:5'-CACGGACCACACTGTCAATC-3'
HO-1	F:5'-GCTGAGAATGCC GAGTTCAT-3'
	R:5'-TGTAGACCGGGTTCTCCTTG-3'
NQO1	F:5'-TGCCTTCCTTGACTTGCT-3'
	R:5'-TCCCGGCTTTACATCCTA-3'
SOD1	F:5'-CAGGTCCTCACTTCAATCC-3'
	R:5'-CCAAACGACTTCCAGCAT-3'
IL-6	F:5'-TACCCCCACGAGAAGATTCC-3'
	R:5'-GCCATCTTTGGAAGGTTCAG-3'
IL-8	F:5'-ACTGGCTGTTGCCTTCTT-3'
	R:5'-CAGTTCTCTTCAAAAATATCTG-3'
IL-10	F:5'-CTGGCATTGTCATGGACTCT-3"
	R:5-GCGATGATCTTGATCTTCAT-3'
IκB	F:5'-TCAACAACTTCTCCGTGGCGCAATG-3'
	R:5'-AGGAGATGGAGGTGCCCTGGCTAA-3'
P38	F:5'-TCTGTAGGAAATCACACTAGG-3'
	R:5'-GTATGCACTTCAGATCTTCAC-3'
P65	F:5'-AGTACCCTGAGGCTATAACTCG-3'
	R:5'-TGAGAAGTCCATGTCCGCAAT-3'
AKT	F:5'-TCAACAACTTCTCCGTGGCGCAATG- 3'
	R:5'-AGGAGATGGAGGTGCCC TGGCTAA-3"
HAT1	F: 5'- GATGATGAA AGATGGCACTAC-3'
	R: 5'-CCTTGACCTTGAAATGGAGT-3'
HDAC1	F: 5'- CCCCAGGGACTAGACAGGAA-3'
	R: 5'- TGGAGAGGGATGGATGGTG-3'
